# Neurapraxia in patients with trigeminal neuralgia but no identifiable neurovascular conflict during microvascular decompression: a retrospective analysis of 26 cases

**DOI:** 10.1186/s12893-022-01469-3

**Published:** 2022-01-11

**Authors:** Juan Li, Min Zhou, Yuhai Wang, Sze Chai Kwok, Jia Yin

**Affiliations:** 1 Department of Neurosurgery, Shanghai Tenth People’s Hospital, Tongji University, No. 301 Yanchang Road, Shanghai, 200072 China; 2 Department of Neurosurgery, Bengbu First People’s Hospital, No. 229 Tushan Road, Bengbu, 23000 Anhui China; 3 Department of Neurosurgery, 904 Hospital of PLA, No. 101 North Xingyan Road, Wuxi, 214044 Jiangsu China; 4 Shanghai Key Laboratory of Brain Functional Genomics, Key Laboratory of Brain Functional Genomics Ministry of Education, School of Psychology and Cognitive Science, East China Normal University, No. 3663 North Zhongshan Road, Shanghai, 200062 China; 5 Shanghai Key Laboratory of Magnetic Resonance, East China Normal University, No. 3663 North Zhongshan Road, Shanghai, 200062 China; 6 NYU-ECNU Institute of Brain and Cognitive Science at NYU Shanghai, No. 3663 North Zhongshan Road, Shanghai, 200062 China

**Keywords:** Trigeminal neuralgia, Microvascular decompression, Offending vessel, neurapraxia

## Abstract

**Background:**

Microvascular decompression (MVD) is the first choice in patients with classic trigeminal neuralgia (TGN) that could not be sufficiently controlled by pharmacological treatment. However, neurovascular conflict (NVC) could not be identified during MVD in all patients. To describe the efficacy and safety of treatment with aneurysm clips in these situations.

**Methods:**

A total of 205 patients underwent MVD for classic TGN at our center from January 1, 2015 to December 31, 2019. In patients without identifiable NVC upon dissection of the entire trigeminal nerve root, neurapraxia was performed using a Yasargil temporary titanium aneurysm clip (force: 90 g) for 40 s (or a total of 60 s if the process must be suspended temporarily due to bradycardia or hypertension).

**Results:**

A total of 26 patients (median age: 64 years; 15 women) underwent neurapraxia. Five out of the 26 patients received prior MVD but relapsed. Immediate complete pain relief was achieved in all 26 cases. Within a median follow-up of 3 years (range: 1.0–6.0), recurrence was noted in 3 cases (11.5%). Postoperative complications included hemifacial numbness, herpes labialis, masseter weakness; most were transient and dissipated within 3–6 months.

**Conclusions:**

Neurapraxia using aneurysm clip is safe and effective in patients with classic TGN but no identifiable NVC during MVD. Whether this method could be developed into a standardizable method needs further investigation.

## Background

Microvascular decompression (MVD) is the first choice in patients with classic trigeminal neuralgia (TGN) that could not be well controlled by pharmacological treatments [1, 2]. However, in 3.1–28.8% of the cases, offending vessels (OVs) could be identified during MVD [3, 4]. In such cases, second surgical operation that ablates the extracranial segment of the trigeminal nerve may be needed. Revuelta-Gutierrez et al. reported promising results of neurapraxia with bipolar tips during MVD in such patients [5]. In 2014, we attempted to develop a standardizable method of neurapraxia by using a Yasargil temporary titanium aneurysm clip (force: 90 g). In the initial series of 3 cases, the sensory root or the main trunk of the trigeminal nerve was clipped for 2.5 min, but we observed severe hemifacial numbness and masseter weakness in all 3 cases. Starting from the beginning of 2015, we decreased the clipping duration to 40 s. In this retrospective analysis, we analyzed the data of all cases with at least 1-year follow-up.

## Methods

The current study was conducted in compliance with the principles outlined in the Declaration of Helsinki, and was approved by the Ethics Committee of Shanghai Tenth People’s Hospital (approval #: CPPRB1). Informed consent was waived due to the nature of the study. All patient data were anonymized in the paper. We retrospectively screened all cases of MVD for TGN at our center during a period from January 1, 2015 to December 31, 2019. The diagnosis of TGN was established based on the criteria by the International Classification of Headache Disorders 3 (ICDH-3; 13.1.1.1). All subjects received MR-angiography prior to the surgery. MVD was performed using a standard suboccipital retrosigmoid approach. After releasing cerebrospinal fluid (CSF) under the microscope, the cerebellar hemisphere was retracted, and the arachnoid membrane between the petrosal vein and the facial-auditory nerve was opened to expose the cisternal segment of the trigeminal nerve. The entire length of the trigeminal nerve root (from the pons to the entrance of Meckel’s cave) was dissected to identify NVC. Teflon fragments were applied between the trigeminal nerve and offending vessels.

**Fig. 1:**
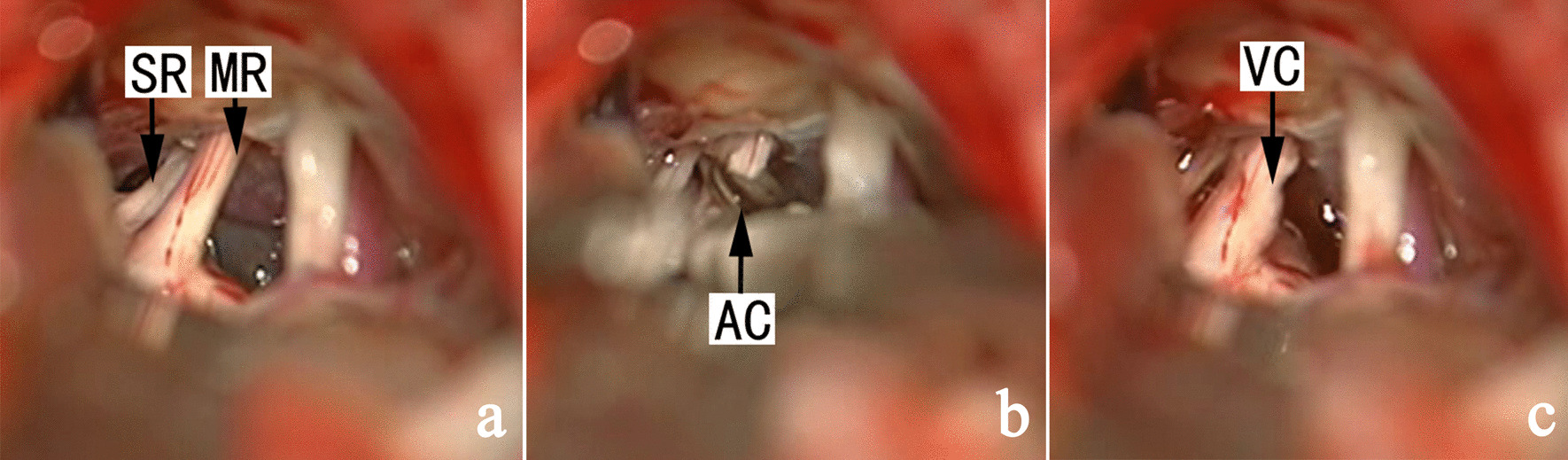
A representative case with no OVs and separate sensory vs. motor trigeminal nerve root. SR: sensory root of trigeminal nerve, MR: motor root of trigeminal nerve, AC: aneurysm clip, VC: vestige of clamp at the sensory root of trigeminal nerve

**Fig. 2:**
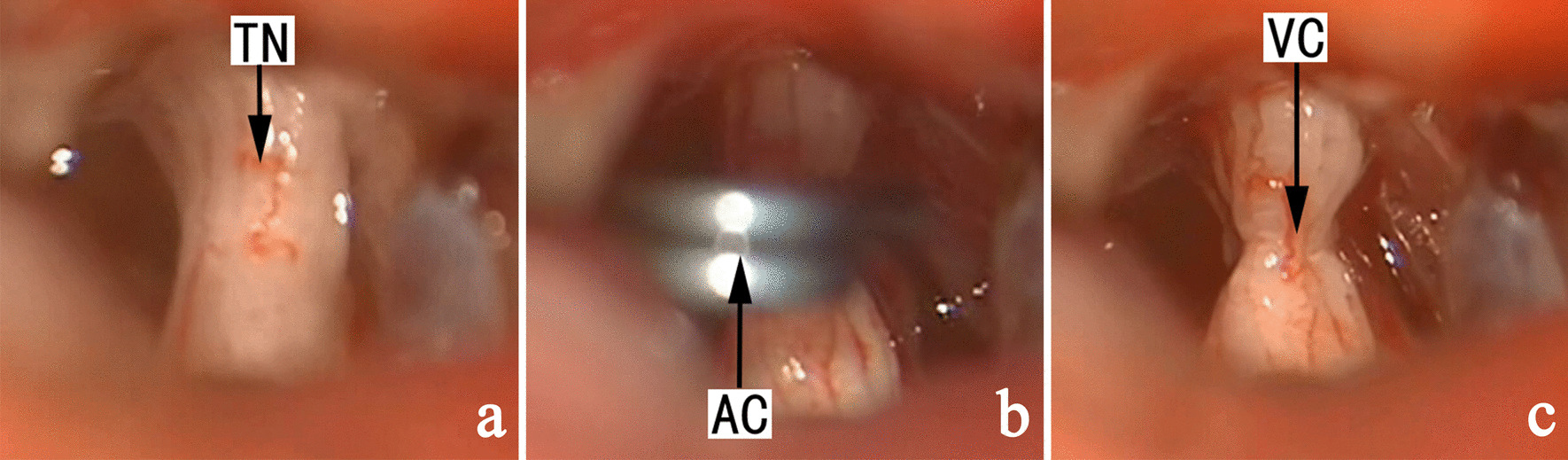
A representative case with no OVs and united sensory and motor roots. TN: trigeminal nerve, AC: aneurysm clip, VC: vestige of clamp at the whole trigeminal nerve

**Fig. 3:**
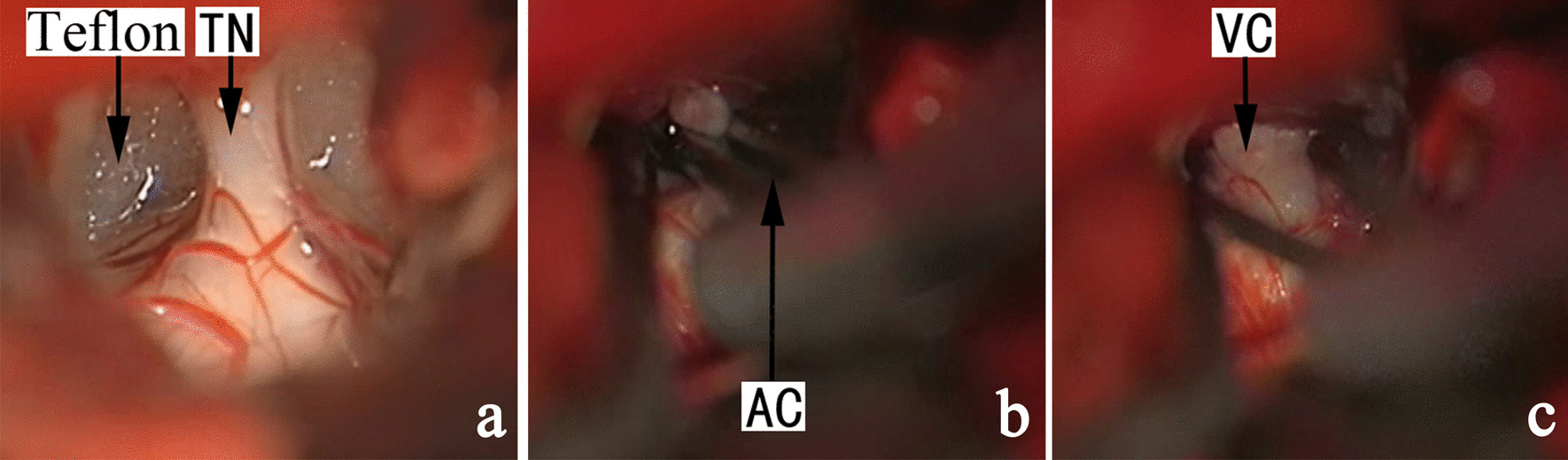
A representative case of recurrent TGN, with no OVs and united sensory and motor root. TN: trigeminal nerve, AC: aneurysm clip, Teflon: Teflon pad, VC: vestige of clamp at the whole trigeminal nerve

In cases without identifiable NVC, neurapraxia was conducted using a standard straight Yasargil temporary titanium aneurysm clip (Aesculap-B. Braum, Germany; 90-g force) for 40 s, preferably to the sensory root (Fig. 1), or the main trunk of the trigeminal nerve if sensory root was not separate from the motor root (Fig. 2). The procedure could be readily conducted in patients with recurrent pain after previous MVD (Fig. 3). In subjects with clinically significant bradycardia or hypertension during neurapraxia, the aneurysm clip was released temporarily and the process was repeated to achieve 60-s accumulative time for neurapraxia before. Sutures were removed 7 days later. The last follow-up (via either office visit or telephone) was conducted in December 2020.

## Results

A total of 26 subjects (median age: 64 years; 15 women) were included in the analysis. Demographic and clinical characteristics are shown in Table 1. Among the 26 patients, 5 received previous MVD for TGN. The median disease duration was 3.25 years (range: 0.5–14.0). The most common territory was V2 + V3. The Barrow Neurological Institute (BNI) pain scores were IV in 9 cases, and V in the remaining 17 cases [6].

**Table 1:** Demographic and baseline characteristics of the patients

	Cohort size (n = 26)
Female sex, no. (%)	15 (57.7%)
Age (y), median (range)	64 (33–81)
Disease duration (y), median (range)	3.25 (0.5–14.0)
Affected side, no. (%)	
Right only	14 (53.8%)
Left only	12 (46.2%)
Territory involved	
V1	1
V2	3
V3	5
V1 + V2	2
V2 + V3	12
V1 + V2 + V3	3

In 16 out of the 26 cases, neurapraxia was completed in one session. In the remaining 10 cases, the process had to be temporarily suspended and repeated due to bradycardia (n = 7) or hypertension (n = 3). No severe peri-operative complications (e.g., intracranial hemorrhage, intracranial infection and cerebrospinal fluid leakage) occurred. Post-operative complications included transient hemifacial numbness (n = 26; 100%), herpes labialis (n = 9; 34.6%), masseter weakness (n = 8; 30.7%), hemifacial formication (n = 2; 7.7%) and blunted corneal reflex (n = 2; 7.7%) (Table 2). Majority of the complications lasted for 3–6 months and eventually dissipated, but facial formication persisted until the last follow-up in both cases (2 and 3 years from the surgery, respectively).

**Table 2:** Post-operative complications

Complications	Cohort size (n = 26) (%)
Hemifacial numbness	26 (100)
Herpes labialis	9 (34.6)
Masseter weakness	8 (30.7)
Hemifacial formication	2 (7.7)
Blunted corneal reflex	2 (7.7)

Immediate and complete pain relief (BNI pain score of I without any medication) was achieved in all 26 cases. Within a median follow-up of 3.0 years (range: 1–6 years), recurrence occurred in 3 (11.5%) patients. The time from surgery to relapse was 1, 1, and 1.5 years, respectively. The BNI pain score was II, II and III with carbamazepine treatment in the 3 patients with relapse. The remaining 23 patients were medication-free.

## Discussion

Vascular compression of the trigeminal nerve root is the leading cause of classic TGN. Other causes include focal arachnoid thickening, adhesion, cerebello-pontine angle tumors, inflammation, multiple sclerosis, brainstem infarction, and arteriovenous malformations [7, 8]. In patients who did not respond to or could not tolerate pharmacological treatments, MVD is the first choice regardless of the presence or absence of NVC as determined by MRI due to the low sensitivity of MRI in detecting NVC [9]. In a small percentage of the patients, NVC could not be identified despite of complete dissection of entire length of the trigeminal nerve root. In addition, complete dissection of the trigeminal nerve in recurrent cases after previous MVD is often difficult, if not impossible, due to adhesion [10, 11].

Partial sensory rhizotomy (PSR) is one option in patients with no identifiable NVC. In a study of 83 cases, Young et al. reported 15% rate of severe complications (complete loss of sensory function and corneal ulcer) [12]. A literature review of 10,493 patients undergoing a variety of surgical treatments for trigeminal neuralgia also supported the high rate of severe complication with PSR [13]. As a result, PSR has been practically abandoned in clinical practice. Nerve combing is another option in TGN patients with no identifiable NVC, but is associated with relatively high rate of 5-year recurrence (approximately 40%) [14–16].

Percutaneous balloon compression (PBC) at the site of the trigeminal Gasserian ganglion is currently recommended as the first-choice extracranial treatment of TGN [17]. The physiological basis of PBC is the higher sensitivity of the larger pain fibers in the trigeminal nerve to physical damages compared to smaller fibers that transmit other sensory input and the afferent fibers [18]. Despite of the advantage of PBC, MVD is the treatment of choice in patients with TGN regardless of the presence or absence of NVC as determined by pre-operative MRI. In other words, PBC is typically used in patients who failed MVD treatment, and must be conducted separately [3, 4].

Cheng et al. used bipolar electrocoagulation tip to produce neurapraxia in 28 patients without OV. 20 patients (71.4%) achieved immediate complete pain relief. With a median follow-up of 46 months (range: 8–60 months), the recurrence rate was 38.4%, and only 13 patients (46.4%) remained pain-free without medication during the follow-up. Four patients (14.3%) developed permanent facial numbness [19]. Revuelta-Gutierrez et al. used bipolar electrocoagulation tips to produce neurapraxia to the trigeminal nerve root in 21 patients, and achieved immediate complete pain relief in all 21 patients. Recurrence rate was 14.8% at 12–36 months and 43.2% at 48 months. Permanent hypoesthesia was present in 6 patients (28.6%), whereas transient loss of corneal reflex was observed in 1 patient (4.8%). Motor function of the trigeminal nerve was intact in all patients [6].

In the current study, we achieved 100% immediate complete pain relief with acceptable complications in 26 patients with no identifiable NVC during MVD. Within a median of 3-year follow-up, the recurrence rate was 11.5%. In our opinion, these encouraging results reflect consistent degree of damage to the sensory fibres of the trigeminal nerve due to the use of consistent force (90 g) and 40-s clipping duration. Whether this method could be developed as a standardizable approach requires further study in different settings.

From a surgical viewpoint, the trigeminal nerve root must be completely exposed to reveal possible OVs. If possible, only the sensory root should be clipped. Also, duration of the clipping is essential. In a pilot series that consisted of 3 cases, we clipped the trigeminal nerve root for 2.5 min, and unfortunately, all 3 patients developed severe hemifacial numbness and masseter weakness. The protocol that we have been using since 2015 is clipping for 40 s if the procedure could be completely in a single attempt, and for a total of 60 s if the procedure must be suspended temporarily and repeated due to bradycardia or hypertension.

In addition to the retrospective nature, the current study is limited by the relatively small sample size and the relatively short follow-up (median at 3 years). Also, 3 patients who were lost to follow-up were not included in the analysis. It is likely that these 3 patients experienced relapse or complications but chose not coming back to us. This could produce some bias to our results. The follow-up was conducted via telephone in some patients, and not based on office visit, adding another layer of limitation to the current study. Having said that, we believe that the key results are solid since recurrence and majority of the complications are sensory abnormalities without standard objective examinations.

## Conclusions

Neurapraxia using a Yasargil temporary titanium aneurysm clip is safe and effective in patients with classic TGN but no identifiable NVC during MVD. The advantage of this method include: (1) potential wider use since the damage is standardizable; (2) no need to schedule a second surgery.

## Data Availability

The datasets used and/or analysed during the current study are available from the corresponding author on reasonable request.

## Abbreviations

MVD: Microvascular decompression
TGN: Trigeminal neuralgia
OVs: Offending vessels
CSF: Cerebrospinal fluid
BNI: The Barrow Neurological Institute
PSR: Partial sensory rhizotomy
PBC: Percutaneous balloon compression
